# Supplementary figures and images for: Characterizing rhizome bud dormancy in Polygonatum kingianum: Development of novel chill models and determination of dormancy release mechanisms by weighted correlation network analysis
Source: PLoS One. 2020 Apr 30;15(4):e0231867. doi: 10.1371/journal.pone.0231867 (PMC7192456; doi:10.1371/journal.pone.0231867)

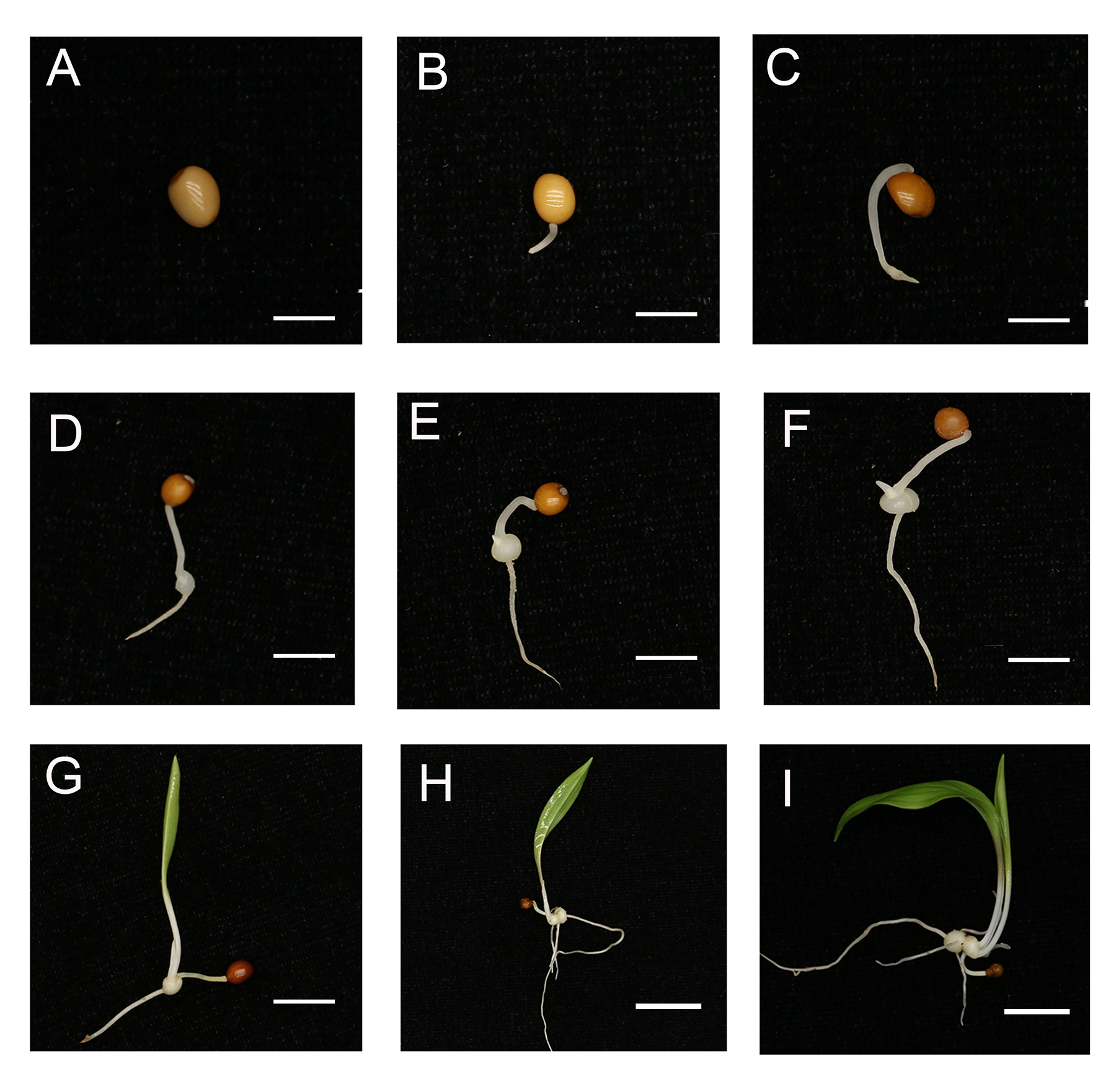

Supplement: S1 Fig — (A) The seed of P. kingianum. (B) Three days after the hypocotyl breaks through the seed coat. (C) The epicotyl begins to swell into a rhizome. (D-F) The rhizomes continue to swell, and roots (D) and buds (F) have formed. (F) The rhizome bud ceases growth and enters endodormancy. (G-I) The rhizome bud grows into a seedling after dormancy is released. (Wang et al. 2019). (TIF) [file pone.0231867.s001.tif]

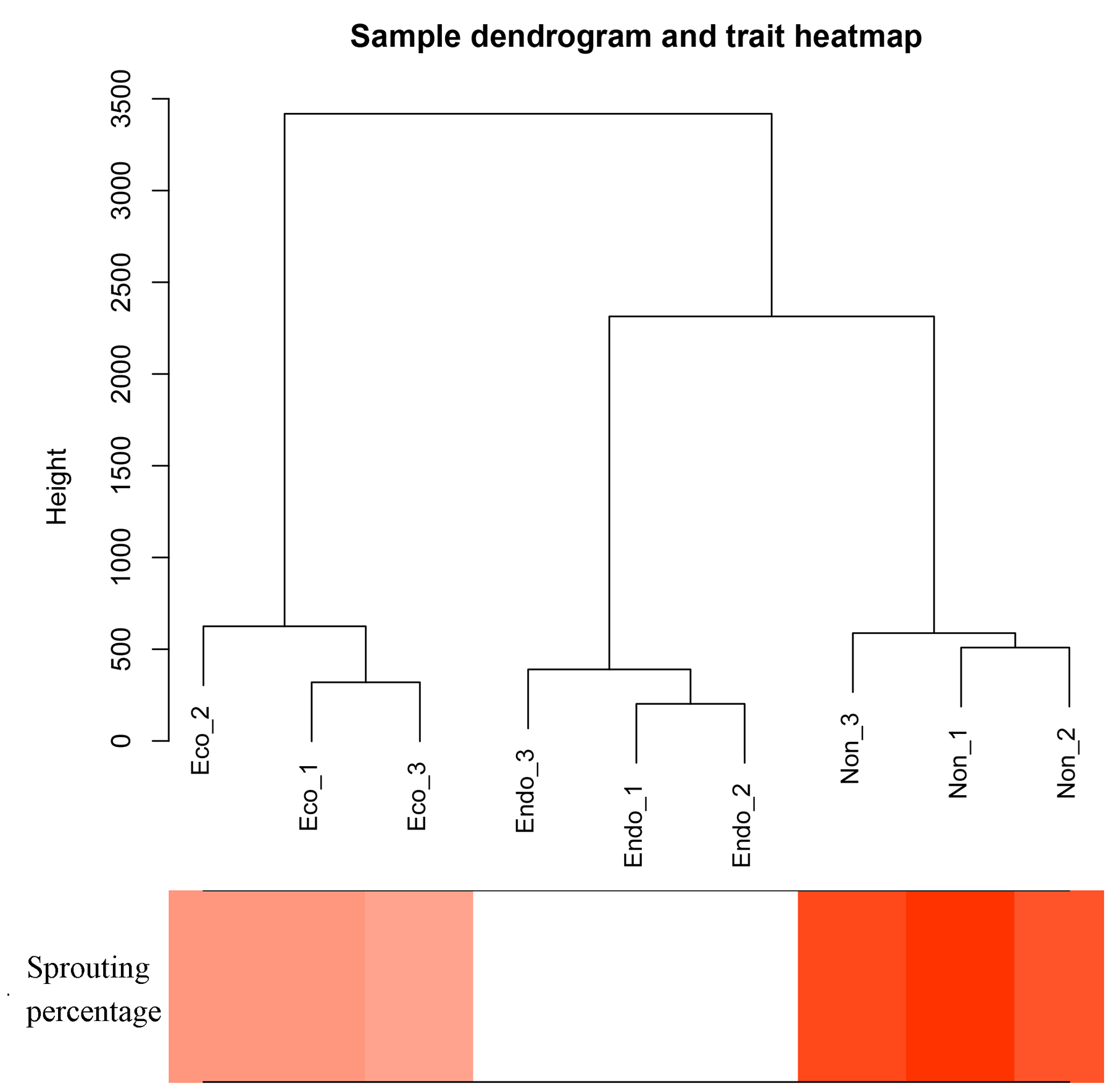

Supplement: S4 Fig — (TIF) [file pone.0231867.s004.tif]

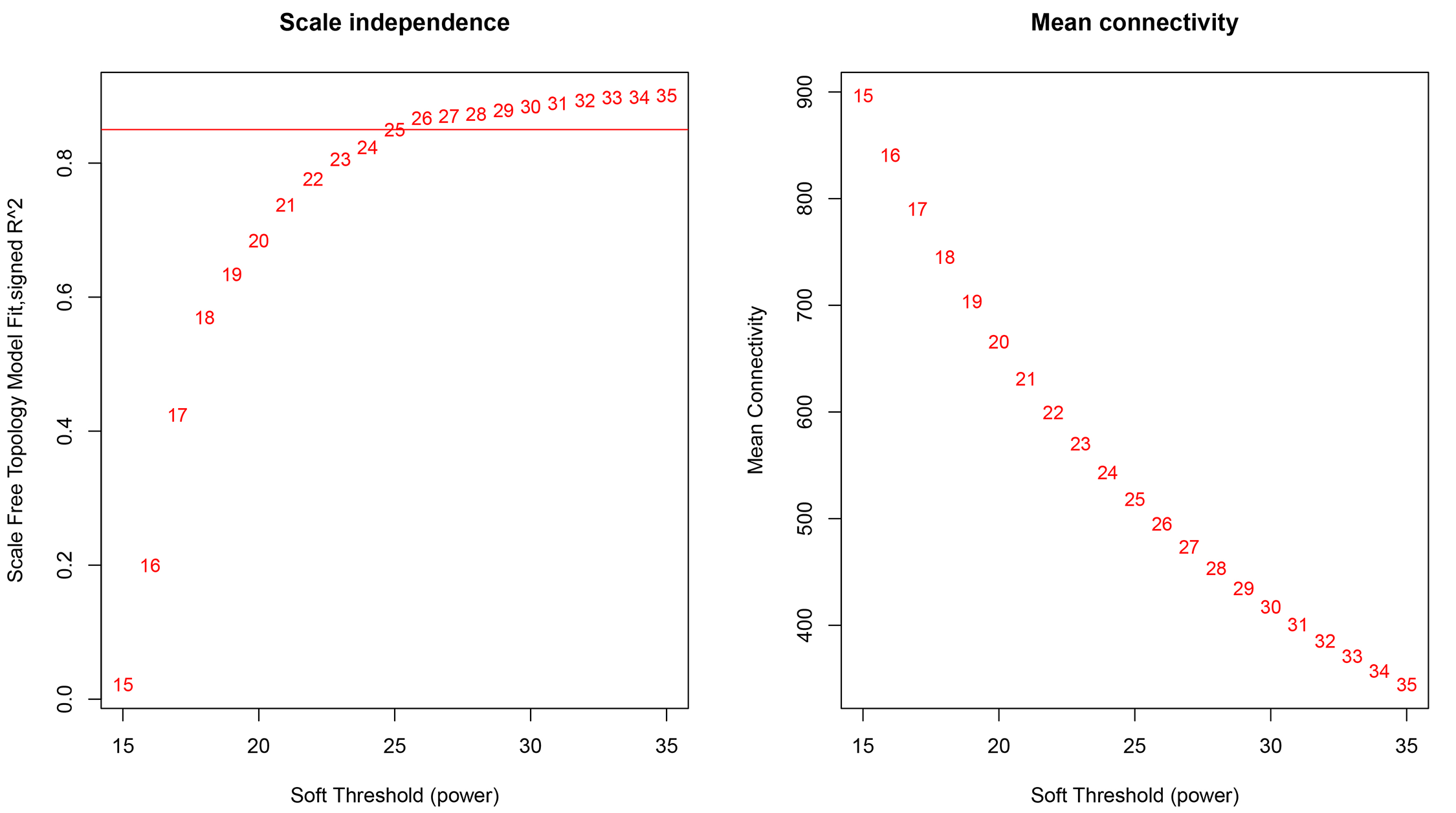

Supplement: S5 Fig — (TIF) [file pone.0231867.s005.tif]

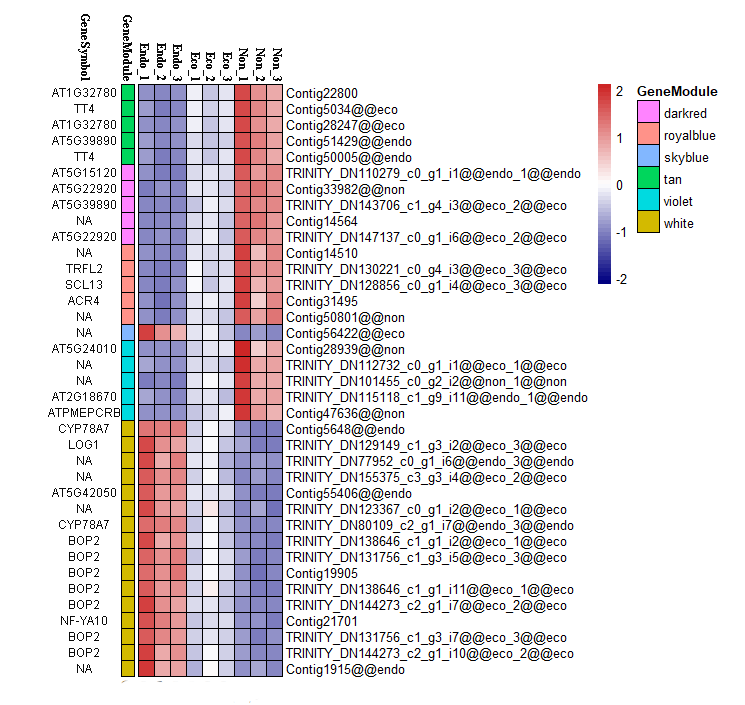

Supplement: S6 Fig — For each gene, the FPKM value normalized by the maximum value of all FPKM values is shown. The gene symbol for each gene is shown on the left. The module attribution for each gene is shown in the second left column and the corresponding module for each color is shown on the right. (TIF) [file pone.0231867.s006.tif]

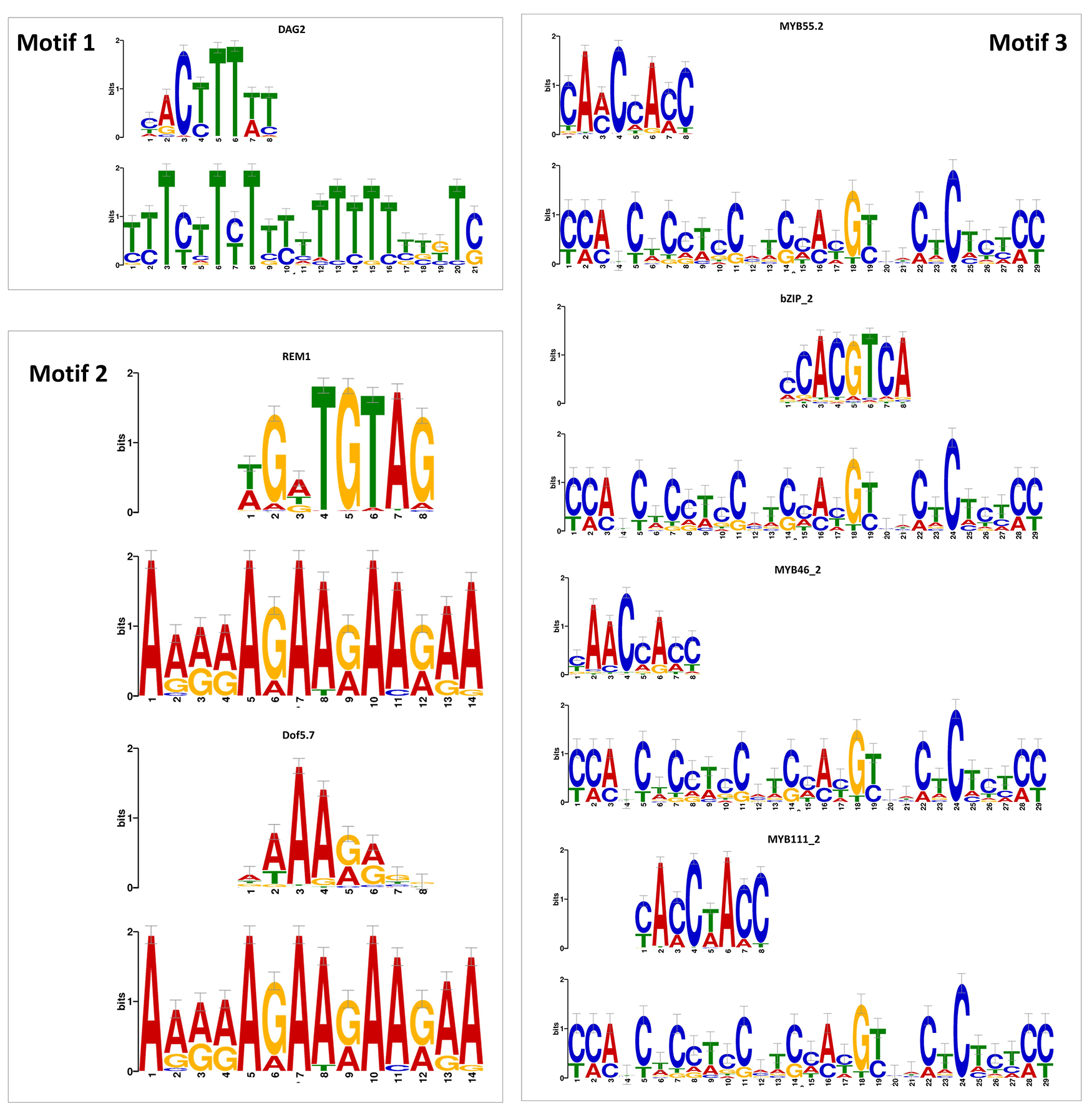

Supplement: S7 Fig — For Motif 1, the TF DAG2 was significantly matched; for motif 2, two significantly-matched TFs, REM1 and Dof5.7, were found; for motif 3, four significantly-matched TFs, MYB55.2, bZIP_2, MYB46_2 and MYB111_2, were found. (TIF) [file pone.0231867.s007.tif]
