# Supplementary material for: Characterizing rhizome bud dormancy in Polygonatum kingianum: Development of novel chill models and determination of dormancy release mechanisms by weighted correlation network analysis
Source: PLoS One. 2020 Apr 30;15(4):e0231867. doi: 10.1371/journal.pone.0231867 (PMC7192456; doi:10.1371/journal.pone.0231867)
Supplement: S4 Table — Through the alignment for the 17 promoter sequences, a total of 3 significantly-matched motifs were found. (DOCX) [file pone.0231867.s011.docx]

**Motif1**

| **Sequence**  **number** | **Sequence name** | **Strand** | **Start site** | ***P*-value** |  | **Motif location** |  |
| --- | --- | --- | --- | --- | --- | --- | --- |
| 4. | gi\|240254678\|ref\|NC_003071.7\|:c17243204-17240204 | + | 418 | 5.31e-11 | **AGAGAAATAG** | **CTTCTTCTTTTTTTTTTTGTC** | **AAAATAATTA** |
| 13. | gi\|240256493\|ref\|NC_003076.8\|:7662142-7665142 | - | 1964 | 1.79e-10 | **AATGGGTTAG** | **TTTCTTTTTCTTTTTTTGGTC** | **AACCAAAATG** |
| 17. | gi\|240256493\|ref\|NC_003076.8\|:8110909-8113909 | - | 2372 | 6.37e-10 | **TAGAAGTACT** | **CTTCTTCTTCTTTTTTTCTTC** | **TTTTTTCTGA** |
| 9. | gi\|240254421\|ref\|NC_003070.9\|:c11875596-11872596 | + | 750 | 8.58e-10 | **TCAAAACTGA** | **TTTCTTTTCTTTTTTTTTGTC** | **GGAATAAAAA** |
| 12. | gi\|240256493\|ref\|NC_003076.8\|:4895813-4898813 | - | 64 | 2.55e-9 | **ATTTAAACAA** | **TCTCTTCTTTTTTTTTCCTTG** | **TTTTCTTAAG** |
| 3. | gi\|240256243\|ref\|NC_003075.7\|:1029478-1032478 | - | 1057 | 7.31e-9 | **TAGTACGCAT** | **TTTTCTCTTTTTTTTTTTGTC** | **AACATATTTT** |
| 14. | gi\|240254421\|ref\|NC_003070.9\|:c2324049-2321049 | - | 2249 | 1.03e-8 | **AAGACCTGTT** | **CTTCTTCTCCCTTCTTGGGTC** | **ACCACCACTG** |
| 5. | gi\|240256493\|ref\|NC_003076.8\|:1982437-1985437 | + | 2093 | 1.38e-8 | **TGAAAGGAGT** | **TTTTTTTTTTTTTTTTTTGTG** | **TTTCAACTTT** |
| 2. | gi\|240254678\|ref\|NC_003071.7\|:8090468-8093468 | + | 2979 | 1.75e-8 | **AGAGGAGAAT** | **TCTTTTTTTTTTTTTTCTTTC** | **CG** |
| 16. | gi\|240254421\|ref\|NC_003070.9\|:25954842-25957842 | + | 451 | 1.96e-8 | **TTCCAACCTT** | **TTTTTTCTTTTTTTTTTGTTG** | **TCTTCAAAAT** |
| 10. | gi\|240256493\|ref\|NC_003076.8\|:4485761-4488761 | + | 2869 | 4.97e-8 | **CCTACCACAC** | **TCTCATCTTCTTTTTCCCGTG** | **TCAGTTTGTT** |
| 15. | gi\|240256243\|ref\|NC_003075.7\|:c9665808-9662808 | + | 2168 | 6.43e-8 | **GTAGTCACTT** | **TCTCCTTTTTATTTTCTTGTC** | **AAAACGATTC** |
| 7. | gi\|240256493\|ref\|NC_003076.8\|:16812629-16815629 | - | 469 | 6.43e-8 | **ACTCCATTAT** | **CTTCTTCTCTCTCTTTCCCTC** | **ATCTCATTAA** |
| 8. | gi\|240254678\|ref\|NC_003071.7\|:12078185-12081185 | + | 2366 | 2.08e-7 | **GTTATTTTAG** | **TCTCTTCTTCGTTTTCGGTCC** | **TCAAACTCGA** |
| 1. | gi\|240256493\|ref\|NC_003076.8\|:c3433143-3430143 | + | 651 | 2.63e-7 | **ATTTCAACGC** | **TTTCGTTTTTTTTTGTTTCTG** | **TTTTCCTTGA** |
| 11. | gi\|240256493\|ref\|NC_003076.8\|:15971542-15974542 | + | 2884 | 5.70e-7 | **TCCCGAAACT** | **GTTCTTTTGCCCTCTTCTCTC** | **AATTTTAATC** |
| 6. | gi\|240256493\|ref\|NC_003076.8\|:3109240-3112240 | - | 2960 | 1.34e-6 | **GATGTACTTT** | **TTTTTTTTGTAATCTTGTTTC** | **TTGTCAAATA** |

**Motif2**

| **Sequence**  **number** | **Sequence name** | **Strand** | **Start site** | ***P*-value** |  | **Motif location** |  |
| --- | --- | --- | --- | --- | --- | --- | --- |
| 6. | gi\|240256493\|ref\|NC_003076.8\|:3109240-3112240 | + | 2600 | 5.78e-16 | **TTCATGCGAC** | **CCACCTCCTCCCTCCACGTGAATCTCTCC** | **CAATTTTAAC** |
| 5. | gi\|240256493\|ref\|NC_003076.8\|:1982437-1985437 | - | 692 | 4.98e-12 | **GGCATCGTCT** | **TCAGCACCACCGGCCATGCTGCTCTCTCC** | **TTTATAGGTC** |
| 17. | gi\|240256493\|ref\|NC_003076.8\|:8110909-8113909 | - | 1710 | 1.38e-11 | **CTGTCCCCCA** | **CAATCTCATCGCTCCACGTGTCTCCTTCC** | **CGCCACCACT** |
| 11. | gi\|240256493\|ref\|NC_003076.8\|:15971542-15974542 | + | 1241 | 4.42e-11 | **GACGAGATAT** | **CCATCTCCTCCGTCACTGTCCACCACCAC** | **TGCTTTTCAC** |
| 4. | gi\|240254678\|ref\|NC_003071.7\|:c17243204-17240204 | + | 796 | 3.24e-10 | **ATCCGGCCGA** | **CACGCTTCTCCATGTCCGTCCCACTCTCT** | **CTCCCATCCA** |
| 15. | gi\|240256243\|ref\|NC_003075.7\|:c9665808-9662808 | + | 757 | 4.60e-10 | **CCGTACAAAT** | **CCCCTACGCGCCTCCACGCGTCGCTTCCT** | **CACTCCCGTC** |
| 10. | gi\|240256493\|ref\|NC_003076.8\|:4485761-4488761 | + | 1009 | 5.02e-10 | **CGGTATAACA** | **CCACCACGAGCCTGTAAGCTACTCTCAAC** | **ATCACTCAAA** |
| 3. | gi\|240256243\|ref\|NC_003075.7\|:1029478-1032478 | + | 1576 | 9.78e-10 | **TTTTACTTCC** | **TCCTCCCCTCCAAGAATGTAACACTCTCT** | **GTCTTTTAGC** |
| 1. | gi\|240256493\|ref\|NC_003076.8\|:c3433143-3430143 | - | 2213 | 4.48e-9 | **TTCTCAAACG** | **TCAACAGCTTCATCAAAGCCTCTCTATCC** | **TACAGTCGAA** |
| 12. | gi\|240256493\|ref\|NC_003076.8\|:4895813-4898813 | + | 1193 | 6.36e-9 | **ACTGGCATTA** | **CAATCTCCAACTCCCACTTCACTCACCAC** | **AACGATGTAA** |
| 2. | gi\|240254678\|ref\|NC_003071.7\|:8090468-8093468 | + | 855 | 8.34e-9 | **GTATGTAGCA** | **CCAATGCATCGAACCCTGTACCTCCGACC** | **ACAAGCTCGA** |

**Motif3**

| **Sequence**  **number** | **Sequence name** | **Strand** | **Start site** | **P-value** |  | **Motif location** |  |
| --- | --- | --- | --- | --- | --- | --- | --- |
| 14. | gi\|240254421\|ref\|NC_003070.9\|:c2324049-2321049 | + | 1754 | 1.96e-8 | **ACCGACAGAG** | **AAGAAGAAGAAGAA** | **GACGAGCTTC** |
| 1. | gi\|240256493\|ref\|NC_003076.8\|:c3433143-3430143 | + | 731 | 1.96e-8 | **AGCATTGAGG** | **AAGAAGAAGAAGAA** | **AGCTTCATAT** |
| 10. | gi\|240256493\|ref\|NC_003076.8\|:4485761-4488761 | - | 176 | 3.28e-8 | **TTGTTGGTCT** | **AAAGAGAAGAAGAA** | **CCTGAGCATT** |
| 5. | gi\|240256493\|ref\|NC_003076.8\|:1982437-1985437 | - | 2666 | 3.28e-8 | **AGGACACAAT** | **AAAGAGAAGAAGAA** | **TCCAAAATGG** |
| 7. | gi\|240256493\|ref\|NC_003076.8\|:16812629-16815629 | + | 2160 | 6.89e-8 | **AACTGGCTTC** | **AGGAAGAAGAAGAA** | **TTGAAGAATA** |
| 16. | gi\|240254421\|ref\|NC_003070.9\|:25954842-25957842 | - | 1813 | 9.18e-8 | **TTGTTAATAA** | **AGAAAGAAGAAGAA** | **AAACGTAAAT** |
| 15. | gi\|240256243\|ref\|NC_003075.7\|:c9665808-9662808 | - | 2021 | 2.02e-7 | **AATCAGCTTG** | **AGGAAGAAGAAGGA** | **GATTGAGATA** |
| 12. | gi\|240256493\|ref\|NC_003076.8\|:4895813-4898813 | + | 2400 | 2.58e-7 | **TTATCAATGA** | **AAAGAGAAAAAGAA** | **ATAACAAAAA** |
| 17. | gi\|240256493\|ref\|NC_003076.8\|:8110909-8113909 | - | 2934 | 6.56e-7 | **TGATGATATT** | **AAAGAGAAGAAGAG** | **ATGGAGAAAC** |
| 9. | gi\|240254421\|ref\|NC_003070.9\|:c11875596-11872596 | - | 577 | 9.25e-7 | **TCTCAGGTAA** | **AGGAAGAAGAAAGA** | **AGCATCATCA** |
| 11. | gi\|240256493\|ref\|NC_003076.8\|:15971542-15974542 | + | 6 | 1.19e-6 | **AGAAT** | **AAGAAGATGAAGAA** | **AATAGGTCTT** |
| 8. | gi\|240254678\|ref\|NC_003071.7\|:12078185-12081185 | - | 1887 | 2.01e-6 | **ATATCTTCAA** | **AAGAAAAAAAAGAA** | **TGTGTTAGTG** |
| 4. | gi\|240254678\|ref\|NC_003071.7\|:c17243204-17240204 | - | 352 | 2.01e-6 | **TGAGAAAAAA** | **AAAGAGAAAAAAGA** | **ATGACAACAC** |
| 6. | gi\|240256493\|ref\|NC_003076.8\|:3109240-3112240 | - | 639 | 2.31e-6 | **TCAATTGGTC** | **AAAGAAAAGAAAAA** | **AGTGTGAATG** |
| 2. | gi\|240254678\|ref\|NC_003071.7\|:8090468-8093468 | - | 1431 | 4.11e-6 | **AATAATATCC** | **ACAAAGAAAAAAAA** | **AATGAACTTT** |
| 3. | gi\|240256243\|ref\|NC_003075.7\|:1029478-1032478 | + | 99 | 4.71e-6 | **TAAAATCAGC** | **AAAAAGAAAACAAA** | **ACATACTATA** |
| 13. | gi\|240256493\|ref\|NC_003076.8\|:7662142-7665142 | + | 1342 | 5.31e-6 | **AGCCTAGTTC** | **AAAAAAAAAAAAAA** | **ATTGTTACTT** |
